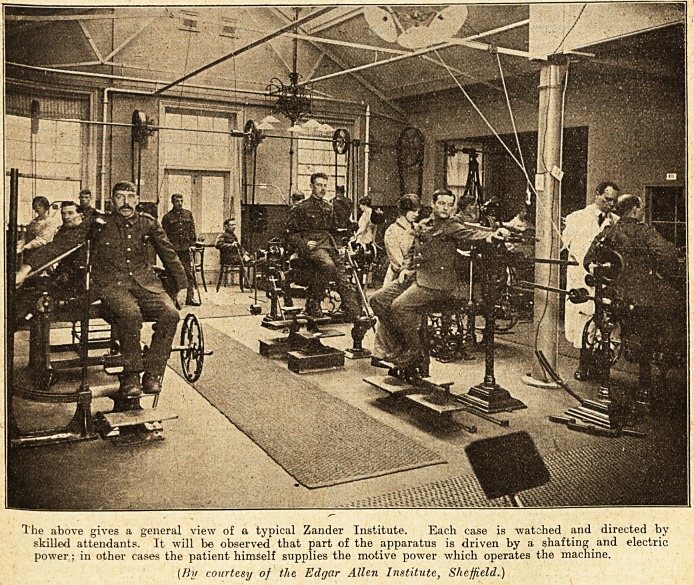# Zander Mechanotherapy

**Published:** 1916-11-25

**Authors:** Gust. Hamel


### PHYSICAL TREATMENT OF WAR INJURIES.

### Zander Mechanotherapy.

By GUST. HAMEL, M.D., M.R.C.S., ETC., M.V.O.

THE excellent organisation for the medical care of the wounded from the trenches to the hospitals and to convalescence, ending with the return to the Front of those who recover, is of course widely appreciated. There is, however, one weak link in this chain and that is the large number of men discharged as disabled and unfit for further service. According to statements in Parliament early this summer, 10,243 soldiers had then been discharged as disabled for injuries to the limbs not necessitating amputation.

The above gives a general view of a typical Zander Institute. Each case is watched and directed by skilled attendants. It will be observed that part of the apparatus is driven by a shafting and electric power; in other cases the patient himself supplies the motive power which operates the machine.

(*By courtesy of the Edgar Allen Institute, Sheffield.*)

There is no doubt these figures could be greatly reduced and many more men made fit again for active work by a more systematic use of modern mechanotherapy.

Massage is freely employed in hospitals and movements are given to injured limbs, sometimes by hand and sometimes by mechanical devices; but strangely enough the most effective form of mechanotherapy, known as Zander mechanotherapy, represented in this country by my own installation in London, the Edgar Allen Institute in Sheffield, and the Zander Institute in Bath, is officially hardly used here at all except at the Croydon War Hospital, the Chatham Naval Hospital, and at a few Red Cross hospitals, and there only at the special request of individual surgeons. In France, on the other hand, there are large installations in the Grand Palais Hospital and in other hospitals, and also ten large private institutions which are now almost exclusively used for the wounded.

A committee of the section of Balneology and Climatology of the Royal Society of Medicine has investigated the methods of treatment adopted for the wounded and disabled soldiers by the French Government, and has reported that great importance is attached to a combination of treatment by "*eau courante*" or whirlpool baths and

Zander mechanotherapy. The report as published in the *Lancet* of February 5, 1916, says:—

"For many cases a complete restoration of function has been obtained, and where this is impossible the figure of incapacity has been considerably reduced, indicating a partial cure of disablement and a substantial economy to the State. The cases completely cured are stated to form 51 per cent. of the whole number treated. Instructive also is the report of the Zander Institute in Sheffield. This Institute was given to the town by the late Sir Edgar Allen some years ago for the free treatment of injured workmen.

Apart from the enormous saving to the State and apart from getting so many more men for active service, there is the future to be considered of those who now remain unfit and unable to gain a decent livelihood. From a humane point of view it is only right that everything should be done with all the means at our disposal to help these men, to restore as much as possible the mobility of their disabled limbs, and to improve thereby their further outlook on life.

### The Development of Zander Treatment in England.

That these aims can best be obtained by a more systematic use of the Zander method of treatment cannot be denied in face of the results obtained both here and abroad, and the fact that this method is nevertheless not more widely used in England is probably due to the conservative tendencies of the medical profession, which as a whole is not yet fully aware of the value of physical methods, and does not therefore sufficiently support and advise the authorities in the matter.

Although there are now Zander installations in practically all modern hospitals and health resorts on the Continent, many years passed by before the medical profession there began to appreciate the treatment. As early as 1857 Dr. Zander, a Swedish physician, conceived the idea of substituting mechanical power for that of the hand in carrying out Ling's system of Swedish gymnastics, but it was not until about 1880 that it became widely popular. He devised three sets of machines. One set, for active movements, is regulated by sliding weights and graduated levers, and is used for exercises from zero to the maximum. The second set, for passive movements, is actuated by motor power; it exercises any part of the body without any exertion on the part of the patient himself. The third set, also connected with motor power, is intended for mechanical operations, such as percussions and vibrations.

The advantage of this system over others is that if the patient is in the right position he must necessarily do the exercises in the proper and effective way. Any change in his position is easily noticed and corrected by the attendant, who can thus superintend a great many patients at the same time. As the amount of weight and range of movement can be easily adjusted, the actual exertion of the patient can be accurately determined and supervised, and all undue strain can be entirely avoided.

The effect of the treatment is no longer dependent on the disposition of an operator, but is more subject to the patient's own will and control. This explains the fact, of great importance in the treatment of many nerve disorders, that Zander graduated exercises have a great recuperative power on the mind and the nervous system, and that they can be applied with great advantage to re-educate muscles and nerves to co-ordinate action. The various forms of mechanical operations, such as vibration, friction, kneading, percussion, etc., as well as all the passive movements, form a very important part of the treatment, and cannot be obtained in any other way. The whole treatment is more attractive to patients, as they can see on the scale the daily or weekly increase of muscular power and of flexibility in the case of a joint. Moreover, patients usually resist any attempt at forcing a joint by hand for fear of being hurt, whereas they have confidence and co-operate with a machine which they know cannot suddenly increase its adjusted range of movement. Gradual mobilisation of a joint in this way has many advantages, and gives better results in the majority of cases than forcible breaking under gas.

### What could be Done in London.

Provided a sufficiently large installation—the machines can now all be made in London—is put up, equal in size to the Grand Palais Hospital in Paris, more than 1,000 men could receive daily treatment; or to deal with a larger number of wounded still, each hospital could have its own smaller installation, if need be, in Y.M.C.A. huts. Properly organised, such installations would require comparatively few attendants, and the enormous amount of time and labour now spent on massage could to a great extent be saved.

Massage is very useful in the earlier stages of an injury, when the tissues and joints are swollen and painful, to remove deposits and effusions, but at a later stage, particularly when adhesions have formed, it is much more important to re-educate the muscles and loosen the contracted joints by carefully graduated passive and active exercises. Passive exercises on the machines act as a form of auto-massage of muscles and joints, which removes effusions and deposits in and about the joint far more effectively than external massage can do. All injuries to legs and arms, necessitating immobilisation of the limb, produce stiffness of the muscles and joints, but it depends on the character of the injury, length of immobilisation, and nature of treatment whether partial or complete restoration of movement or permanent disablement follows. In considering this question we can usefully make the following discrimination:—

I. *Post-operative Stiffness, or Stiffness due to Long Immobilisation.*—This form is usually very painful, but otherwise offers no difficulties and improves readily by a course of graduated exercises on the Zander machines. Stiffness due to immobilisation in the treatment of fracture could, of course, be avoided if massage and movements were applied soon after the injury.

II. *Stiffness due to Injuries to the Joint Itself, Shrapnel or Bullet Wounds, Fractures, etc*—Here, again, stiffness could have been prevented in many cases by early massage and exercises, but my experience is that in at least 75 per cent. of cases, even at later stages, complete restoration of movement can be obtained by mechanotherapy.

III. *Stiffness due to Septic or Rheumatic Conditions.*—Rheumatism is comparatively favourable to treatment, but if sepsis is associated with one of the above-mentioned injuries, it usually leads to strong adhesions in the joint and a matting together of all tissues, so that the chances of perfect recovery are relatively small. There is, however, hardly any case where considerable improvement both in regard to the mobility of the joint and the development of muscular tone and power cannot be effected by perseverance.

### Exercise in Medical Cases.

I have only dealt with joint affections in this paper, as Zander mechanotherapy is of special importance in these cases, and particularly so at the present time; but, of course, there are a great many other conditions in connection with the war, such as soldier's heart, rheumatism, trench feet, gas poisoning, shell shock, etc., in which excellent results can be obtained by utilising the well-known physiological effects of graduated exercises on the circulation, on the heart, or on the nervous system etc. I have also omitted to deal with other forms of treatment as being outside the scope of this paper, but it stands to reason that in order to obtain the best results in each case it is often necessary to apply other forms of treatment as well. There are physical methods which are sedative and others which are more stimulating, and the result of the treatment often depends whether one or another of these methods or a combination of them is judiciously applied to suit each individual case. Particularly useful in joint cases and in conjunction with Zander mechanotherapy are the various forms of dry or moist heat applications to alleviate pain and to prepare stiff joints for the exercises by softening the tissues, but hydrotherapy and electrotherapy have also important functions, and an ideal installation of this kind should, like the Grand Palais Hospital in Paris, provide facilities for all these methods of treatment.

## Figures and Tables

**Figure f1:**